# Sequence analysis of an Archaeal virus isolated from a hypersaline lake in Inner Mongolia, China

**DOI:** 10.1186/1471-2164-8-410

**Published:** 2007-11-09

**Authors:** Eulyn Pagaling, Richard D Haigh, William D Grant, Don A Cowan, Brian E Jones, Yanhe Ma, Antonio Ventosa, Shaun Heaphy

**Affiliations:** 1 Department of Infection Immunity and Inflammation, University of Leicester, University Road, Leicester, LE1 9HN, UK; 2 Department of Biotechnology, University of the Western Cape, Bellville 7535, Cape Town, South Africa; 3 Genencor International B, V., Archimedesweg 30, 2333 CN Leiden, The Netherlands; 4 State Key Laboratory of Microbial Resource, Institute of Microbiology, Chinese Academy of Sciences, Beijing, 100080, China; 5 Department of Microbiology and Parasitology, Faculty of Pharmacy, University of Sevilla, Sevilla, 41012, Spain

## Abstract

**Background:**

We are profoundly ignorant about the diversity of viruses that infect the domain *Archaea*. Less than 100 have been identified and described and very few of these have had their genomic sequences determined. Here we report the genomic sequence of a previously undescribed archaeal virus.

**Results:**

Haloarchaeal strains with 16S rRNA gene sequences 98% identical to *Halorubrum saccharovorum *were isolated from a hypersaline lake in Inner Mongolia. Two lytic viruses infecting these were isolated from the lake water. The BJ1 virus is described in this paper. It has an icosahedral head and tail morphology and most likely a linear double stranded DNA genome exhibiting terminal redundancy. Its genome sequence has 42,271 base pairs with a GC content of ~65 mol%. The genome of BJ1 is predicted to encode 70 ORFs, including one for a tRNA. Fifty of the seventy ORFs had no identity to data base entries; twenty showed sequence identity matches to archaeal viruses and to haloarchaea. ORFs possibly coding for an origin of replication complex, integrase, helicase and structural capsid proteins were identified. Evidence for viral integration was obtained.

**Conclusion:**

The virus described here has a very low sequence identity to any previously described virus. Fifty of the seventy ORFs could not be annotated in any way based on amino acid identities with sequences already present in the databases. Determining functions for ORFs such as these is probably easier using a simple virus as a model system.

## Background

The three domain description of cellular life on earth, *Eukarya*, *Bacteria *and *Archaea *is a firmly established biological tenet [1]. Each domain has an associated, probably vastly diverse, virus population [2-6]. Thousands of viruses infecting representatives of the domain *Eukarya *have been described and many of their DNA/RNA genomic sequences determined [7]. Something like 5–6000 viruses (bacteriophages) infecting representatives of the domain *Bacteria *have been described, at least morphologically, although rather fewer DNA/RNA genomic sequences have been determined [8]. In contrast we are largely ignorant about viruses infecting representatives of the domain *Archaea*. Just 40 or so have been described and the genomic sequences of only a few have been determined, sixteen being listed in Genbank. All archaeal viruses so far discovered have dsDNA genomes, both linear and circular [8,9]. Archaeal viruses having an RNA genome have not yet been identified and *perhaps *do not exist [9].

The domain *Archaea *is divided into four established kingdoms, the *Crenarchaeota*, the *Euryarchaeota*, the uncultivated *Korarchaeota *and the very recently identified *Nanoarchaeota *[10,11]. Virus particles associated with the first two phyla have been identified, recently reviewed in [9]. About 24 viruses of crenarchaeotes have been identified, often with unusual shapes, e.g. droplets and bottle shapes never observed elsewhere; these viruses have no obvious relationship to phage infecting members of the domain *Bacteria *[8,9]. Similarly about 20 viruses infecting members of the *Euryarchaeota *have been identified of which 15 infect haloarchaea, recently reviewed in [12]. These are mostly head/tail viruses of the order *Caudovirales*, including myoviruses and siphoviruses that may be distantly related to those infecting the domain *Bacteria *[8,9]; although other morphotypes have also been observed [12]. Only six viruses of the haloarchaea have been sequenced. All were isolated by the Dyall-Smith laboratory in Melbourne, from hypersaline sources in Australia, except for ***φ***Ch***1***. ***φ***Ch***1***, a temperate myovirus with a 58.5 kb linear genome, the host of which is the haloalkaliphile *Natrialba magadii *[13] was isolated from a laboratory strain and presumably originates, like the host, from Africa. Lytic viruses HF1 and the closely related HF2, having linear genomes of 75.9 kb and 77.7 kb, infect the haloarchaea *Haloferax lucentense *and *Halorubrum coriense *respectively [14,15]. His1 and the distantly related His2 spindle shaped viruses with linear genomes of 14.5 and 16 kb respectively, both have lytic and carrier status in *Haloarcula hispanica *[16]. Finally a lytic icosahedral virus SH1, having a linear genome of 31 kb infects *Har. hispanica *[17,18].

We have been studying both archaeal and bacterial prokaryotic diversity in Chinese salt lakes in Inner Mongolia; as part of this study we looked for virus particles associated with haloarchaea. In this report we describe the complete genomic sequence of a ~43 kb virus BJ1.

## Results

### Description of site and lake water parameters

Lake Bagaejinnor is a hypersaline lake in Inner Mongolia, China [coordinates N45 08 527 E116 36 167]. The lake was sampled in September 2003. It had substantially evaporated over the summer, exposing expanses of [pink salt – encrusted] mud flats and had been reduced to small pools and lagoons of salt – saturated colourless water, pH 8.5. The pink colouration of the salt crystals indicated the presence of haloarchaea. The chemical composition of lake water was determined using laser inductively coupled plasma optical emission spectrometry by the Department of Geology, University of Leicester. Carbonate/bicarbonate concentrations were determined by titration with H_2_SO_4 _using a Digital Titrator Model 16900 according to manufacturer's instructions (Hach Systems for Analysis). Chemical concentrations were Na, 5.32 M; Cl, 4.61 M; S 1.07 M; Mg, 0.35 M; K, 33.25 mM; Br, 8.05 mM; HCO_3_, 7.4 mM; B, 4.25 mM; CO_3_, 3.3 mM; Ca, 0.77 mM; Li, 0.33 mM.

Obviously this is a seasonal chemical analysis of the lake water, the composition of which continually varies, more dilute in spring following the winter thaw and then gradually becoming concentrated by the hot summer winds. We used trial and error techniques to find an appropriate medium where we could pour both top and bottom agars. Medium composition was influenced by very high salt concentrations interfering with agar solidification and causing "salting out" of some of the components. The eventual salt composition of this medium was identical to that determined for the lake above with the following exceptions; Na was at 2.85 M, Cl was at 2.6 M, S was at 0.642 M, Ca and Li were omitted completely.

### Identification of a haloarchaeal host

**Figure 1:**
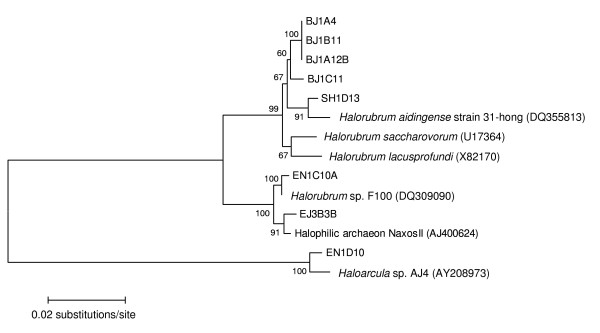
Unrooted phylogenetic tree showing the relationship of the environmental archaeal strain host BJ1B11 for the virus BJ1, to other closely related environmental strains isolated by us and *Halorubrum *species. The scale bar represents the number of inferred nucleotide substitutions per site. Values at nodes indicate >50% percentage occurrence in 500 bootstrapped trees.

Virus BJ1 was isolated from the water column of Lake Bagaejinnor and propagated using strain BJ1 B11. The host was characterised by 16S rRNA gene sequence using both forward and reverse primers, giving 1305 bp of sequence [EMBL: AM412370]. Strain BJ1 B11is most closely related, at 98% identity, to *Halorubrum saccharovorum *with 1289 identical nucleotides. It is also closely related to *Hrr. lacusprofundi *(1283 identical nucleotides) and the recently described *Hrr. aidingense *(1286 identical nucleotides). All three species were originally isolated from hypersaline environments, a salt pan in San Francisco, Deep lake Antarctica and Xin-Jiang in China respectively [19,20]. Fig 1 is a phylogenetic tree showing the relationship of the BJ1 B11 isolate to other closely related sequences present in the BLAST database.

### Plaque morphology

Plaques for BJ1 required one to two weeks to appear on plates because the host is slow growing. Plaque size for BJ1 was variable between experiments ranging from 1–5 mm in diameter, probably due to slight changes in growth conditions; they were also irregularly shaped and turbid. No attempt was made to optimise plaque formation by modifying temperature, salt concentrations or host strain.

### Electron microscopy

**Figure 2:**
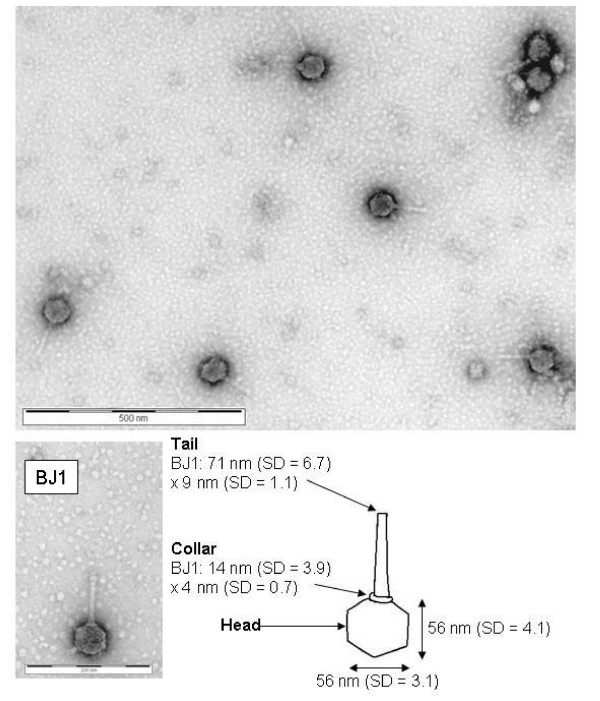
Electron micrograph images of BJ1; the scale bar is 500 nm, top panel and 200 nm bottom panel. A schematic diagram of BJ1 annotated with discernible features and the size of these features is also shown. The standard deviation (SD) of measurements from twenty six different particles was determined.

Virus BJ1 has an icosahedral head, collar and tail, (Fig. 2). The icosahedral head usually has an electron dense shadowing in the centre. The sizes of these features are shown in the schematic diagram Fig 2. The length of a single vertex is 28 nm. The average length of an entire virus particle is about 127 nm. The virus appears to be non-contractile and can be tentatively assigned to the *Siphoviridae *family, (see the Discussion).

### Characterisation of virus genome

**Figure 3:**
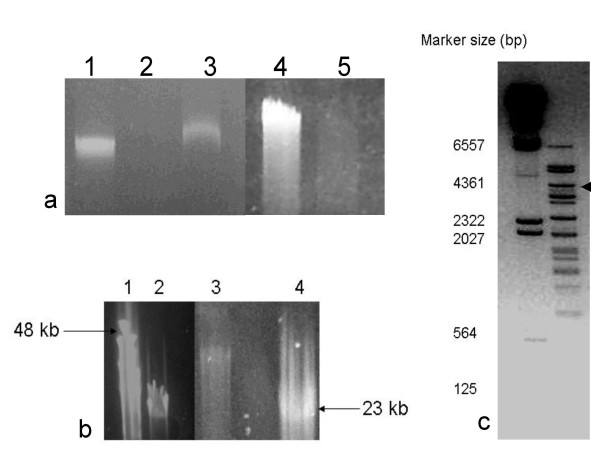
Panel a. 0.8% TAE agarose gel showing virus BJ1 genome sensitivity to nucleases. Lanes 1 and 4 , undigested controls; Lane 2, DNAse treated; Lane 3 RNase treated; Lane 5, exonuclease III treated. Panel b, 1% agarose 0.5× TBE pulse field gel; lanes 1 and 4 size markers (kbps), lanes 2 and 3 BJ1 virus genome. Panel c, *Bam*H1 enzyme digest of virus BJ1 genomic DNA, DNA size markers are shown on the left (kbps). The image has been overexposed to show the smaller bands.

The genomic nucleic acid was tested for susceptibility to various nucleases (Fig 3) Control experiments showed that no virus – associated nucleases were responsible for the degradation observed in these experiments. Fig. 3, lanes 1 and 4 show undigested genome controls, lane 2 shows that the genome was sensitive to DNase I digestion and lane 3 shows that the genome was insensitive to RNAse A. Susceptibility to a wide range of double strand – specific endonucleases i.e *Bam*HI, *Hae*III, *Sst*I and *Xho*I, confirmed that the DNA was double stranded e.g. (Fig 3, panel c). Exonuclease III, specific for linear or nicked circular dsDNA, failed to cut circular double stranded DNA plasmid DNA controls (not shown) but substantially degraded virus genomic DNA (Fig 3 panel a, lane 5). Thus BJ1 probably has a linear dsDNA genome, although the possibility that it is a nicked circular genome cannot be completely ruled out.

Genomic nucleic acid ran on 1.2% TAE agarose gels as a discrete single band larger than a 23 kb DNA marker band. (data not shown). PFGE also suggested a genomic size greater than 23 kb but less than 48 kb (Fig 3, panel b). *Bam*H1 digestion of the genomic DNA gave 21 discrete bands ranging in size from 6.5 kb to ~500 bp (Fig 3, panel c). From the size of these fragments we estimated a genome size of 42.7 kb, remarkably close to the size eventually determined by sequencing (42.271 kb, see below). *In silico *digestion of the determined sequence with *Bam*HI showed that it would generate 20 different fragments i.e. 4949, 4661, 3762, 3235, 3185, 2952, 2434, 2406, 2004, 1949, 1679, 1617, 1505, 1314, 1275, 1094, 816, 781, 563, and 90 bps, with sizes in close agreement to those we observed. Thus the genomic DNA is not subject to methylation at *Bam*HI sites.

### Genome sequence of BJ1

**Figure 4:**
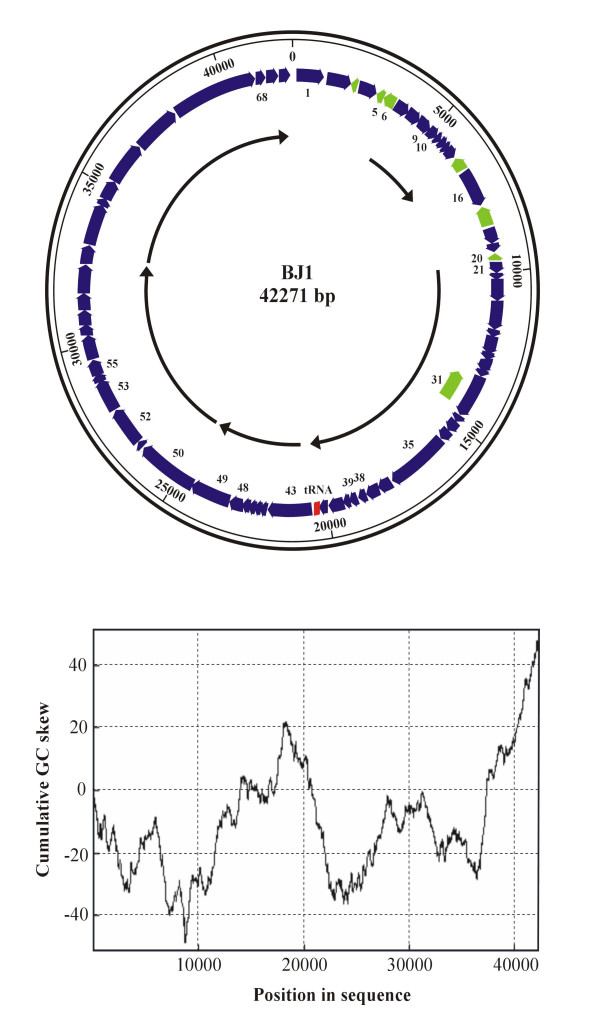
Top panel. Diagram of the BJ1 genome drawn in a circular form. The major features are shown including the predicted ORFs, blue arrows in the forward direction, green arrows in the reverse. The tRNA gene is in red. ORFs mentioned in the text are numbered. The outer scale bar is in base pairs. The inner curved arrows indicate entirely hypothetical operons. The bottom panel shows the cumulative GC skew.

See Figure 4 and Table 1. The double stranded genomic DNA isolated from virus particles is shown as a circular sequence 42, 271 bp long with a G+C content of 64.8 mol% [EMBL: AM419438]. Exonuclease III susceptibility showed that the DNA is linear but sequence assembly indicated it to be circular. This indicates that the genome is terminally redundant (and may be circularly permuted). It is unclear if the BJ1 genome ever forms a circular molecule but if it does then *cos *sites are unlikely to be involved as digests with three infrequent cutting restriction enzymes (*Hind*III, *Eco*RV and *Eco*RI) followed by melting at 80°C failed to show any change in the number of bands compared to un-melted digests (data not shown).

In the absence of an obvious end for the genome from our sequencing experiments we analysed the cumulative GC skew of the sequence (Fig. 4). Skew minima and maxima often represent initiation and termination points of DNA replication in prokaryotes and viruses with a cumulative increase in skew related to the direction of replication and transcription [21]. A clear maximum was observed at about 43000 followed by a sharp change with the minima from 1–8000. This in conjunction with the ORF map and pattern of operons was used to designate a +1 start of the genome (Fig. 4). The cumulative GC skew is consistent with the reading direction of most ORFs and a rolling circle pattern of DNA replication. A single tRNA for phenylalanine (GAA anticodon recognising a UUC codon) was identified using the tRNAscan-SE program. Potential ORFs were assigned using the programs FGENESB and GeneMark.hmm v2.5a (set for prokaryotes); these analyses predicted 63 and 66 ORFs, respectively, encoding polypeptides larger than 30 amino acids. We further analysed the regions upstream and downstream of these predicted ORFs for putative ribosome binding sites and overlapping start and stop codons, and found several additional ORFs. BLAST searches using the amino acid sequences of all predicted ORFs were used to differentiate between possible genes e.g. ORFs 5 and 6 have matches (see below), so putative ORFs in the opposite strand with no BLAST matches have been discounted. By combining all of the data we conclude that BJ1 probably contains 70 ORFs (Fig 4 and Table 1). [If we only count ORFs greater than 60 aa in size then the number of ORFs drops to 55]. Taking the upper estimate of 70 gives an ORF density of 1.65/kb. This is fairly close to the figure of 1.7 ORFs/kb observed for other archaeal virus genomes (17). The majority of the ORFs have initiation codons of ATG (62) and the rest are GTG (8).

**Table 1:** Predicted ORFs in virus BJ1

ORF	Start	Stop	aa	Mr	pI	RBS/distance
**1**	130	990	286	33	4.6	-
**2**	1146	1805	219	25	4.9	-
**3-**	1980	2093	37	3.7	8.5	GGAGGTG-5
**4**	2207	2425	72	8.1	7.0	-
**5-**	2541	3191	216	24	4.7	GAGG-10
**6-**	3178	3393	71	8.2	4.3	-
**7 V**	3547	3993	148	16	4.1	GAG-6
**8**	3993	4463	156	18	4.3	AGGAGGTGA-8
**9**	4456	4851	131	15	4.2	AGGAGGTGA-7
**10**	4844	5218	124	14	4.7	GGAGGT-6
**11**	5208	5357	49	5.2	3.8	GAGGTG-8
**12**	5350	5574	74	8.3	4.6	AGGAGGT-6
**13**	5571	5744	57	6.1	10.4	GGAGG-5
**14**	5741	5986	81	8.9	5.8	GGAGG-8
**15-**	5998	6417	139	15	4.3	GAGG-7
**16**	6637	7713	358	40	5.0	AGGTG-9
**17-**	7919	8560	213	24	4.3	AGGA-8
**18**	8689	8949	86	9.3	4.9	-
**19**	8950	9153	67	7.9	5.2	GGTG-10
**20-**	9159	9446	95	11	4.6	GGAG-4
**21**	9660	10022	120	14	4.6	GGA-7
**22**	10022	10153	43	4.6	4.0	GGTG-8
**23**	10153	10890	245	28	3.9	GGAGG-8
**24**	10880	11806	308	34	4.3	GGAGG-9
**25 V**	11803	11946	47	5.2	4.1	GGTGA-7
**26**	11946	12671	241	27	4.7	GGTGA-7
**27 V**	12668	12760	30	3.3	4.5	GGAGGTG-6
**28**	12757	13092	111	12.2	5.8	GAGGTGA-5
**29**	13092	13262	56	6.2	3.8	GGAGG-8
**30**	13255	14700	481	52	6.2	AGGAGG-6
**31-**	13270	14487	405	46	5.0	-
**32**	14701	14826	41	4.3	4.0	GGAGGTGA-9
**33**	14819	15307	162	18	4.6	GAGGTGA-7
**34**	15310	15531	73	83	11.6	AGGAGGTG-9
**35**	15489	17603	704	78	4.7	(GAAAA)
**36**	17606	18058	150	17	4.4	GGAGG-9
**ORF**	Start	Stop	aa	Mr	pI	RBS/distance
**37**	18055	18519	154	18	4.3	(GGGGG)
**38 V**	18512	18817	101	11	5.0	GAGGTG-8
**39 V**	18814	19074	86	9.9	6.1	GAGGTG-9
**40 V**	19071	19241	56	5.9	10.3	GGAGG-8
**41 V**	19129	19806	225	26	6.3	-
**42**	19803	19982	59	6.4	4.0	GAGGTG-6
**tRNA**	19973	20046	-	-	-	-
**43**	20365	21843	492	55	4.9	-
**44**	21840	21998	52	5.9	4.3	-
**45**	22001	22111	36	3.9	4.8	-
**46**	22108	22416	102	11	4.3	-
**47**	22416	22577	53	6.1	4.2	-
**48**	22574	23083	169	19	4.3	-
**49**	23080	24423	447	50	4.9	GAGG-8
**50**	24427	26382	651	73	4.5	-
**51**	26461	26586	41	4.4	4.4	GAG-9
**52**	26590	27933	447	47.	3.9	AGGAGG-9
**53**	27949	29031	360	40	4.2	GTGA-8
**54**	29040	29219	59	6.4	3.8	GAGGTGA-4
**55**	29222	29572	116	12	3.9	-
**56**	29576	30451	291	33	4.6	GGAGG-9
**57**	30444	30761	105	11	4.1	-
**58**	30758	31210	150	17	4.8	AGG-10
**59**	31207	31734	175	20	4.5	GGAGGT-5
**60 V**	31766	32680	304	32	3.8	GAGGTGA-7
**61**	32680	33177	165	18	4.0	AGGAGGTGA-8
**62**	33281	34408	375	38	4.1	-
**63**	34444	34731	95	10	4.8	-
**64**	34771	35439	222	24	4.0	-
**65**	35446	36633	395	42	4.1	TGA-7
**66**	36634	38226	530	52	3.7	AGGAGGTG-10
**67**	38229	40979	916	100	4.0	GGAGGTG-15
**68**	41059	41400	113	12	3.8	GGAG-6
**69**	41403	41843	146	16	4.6	AGGTG-9
**70**	41840	42151	103	11	3.9	GGTGA-4

Orfs are in the forward direction unless indicated by a -ve sign. v indicates a valine start. aa indicates the number of amino acids. Mr is the molecular mass × 10^-3^, rounded to the nearest 100. pI is the isoelectric point rounded to one decimal place. rbs/distance is the ribosome binding site sequence and its distance from the start codon.

The Shine/Dalgarno sequence from *Halobacterium (Halorubrum) saccharovorum *16S rRNA gene sequence (Accession HSU17364), which is the closest phylogenetic match to the phage host was complemented (AGGAGGUGA) and used to search 5–15 bp upstream of each putative start site for the presence of putative ribosome binding sites (RBS). 51 of the 70 ORFs had sequences suggestive of a RBS, (Table 1). One particular stretch of 6 predicted ORFs (ORF43-ORF48) showed no obvious RBSs at all. A lack of a RBS for some genes is not surprising as archaeal transcription/translation is a mosaic of prokaryotic and eukaryotic mechanisms and the first gene of an operon, or a singly transcribed gene often lacks a RBS [22-24].

The majority of the ORFs (59/70) had a low calculated isoelectric point (pI < 5), which is similar to the acidic proteins of halophilic organisms [15,25]. Just three small ORFs (less than 74 aa) were predicted to be extremely basic (pI > 10). No ORF larger than 100 aa had a pI above 6.3. 63 ORFs and the tRNA are coded on one strand (designated forward) and 7 are on the reverse strand. One ORF, 30 (13255–14700 bp) overlaps entirely with another, ORF31 (13270–14487 bp), running in the opposite direction. It seems probable that both ORFs are coding, ORF30 because it overlaps with the start and stop codons of the ORFs before and after it i.e. 29 and 32, with a good consensus RBS; ORF31 because it shows significant homology to integrases, (see below).

### BJ1 ORF analysis

BlastN analysis of the whole virus genome showed significant matches (E 10^-9 ^to 10^-4^) to small segments of several haloarchaeal sequences i.e. *Natronomonas pharaonis*, *Halobacterium *sp. NRC-1 and *Har. marismortui*. BlastX analysis identified four regions of the genome having significant matches to data-base proteins either from haloviruses or haloarchaea, discussed below. The putative ORFs were individually analysed using BlastX and BlastP. InterPro was also used to search for functional domains. Using these approaches we were unable to ascribe any match or function to 50 of the 70 ORFs i.e. E values were greater than 0.05. Of the 20 we could match i.e. E value less than 0.05, most were to haloarchaeal virus entries or to haloarchaea. These results are summarised in Table 2. Of these 20, 4 were matches to data-base entries with no identifiable function, i.e.: ORF9, ORF10, ORF17, ORF55 and ORF 24.

**Table 2:** BJ1 ORFs with identifiable BlastX matches to data base entries.

ORF	Homologs (% Identity)
9	59% similarity (E 10^-8^) to ORF58 halovirus ***φ***Ch1 (AAM88732)
10	54% similarity (E10^-5^) to protein *Haloquadratum walsbyi *(CAJ52235)
17	similarity (E 10^-13^) to protein from *Natronomonas pharaonis *(CR936257.1)
55	similarity (E 10^-7^) to a protein of ***φ***Ch1 (NP 665930.1)
24	No significant match to any described protein. InterPro suggests DNA binding protein
	
5	similarity (E 10^-3^) to bacterial proteins with DnaJ domain; role in DNA replication?
6	65% similarity (E 10^-6^) to protein (AAG20925) of *Halobacterium *sp. NRC-1. A metal regulated homodimeric repressor with a 'winged helix' DNA binding domain
16	60% similarity (E 10^-67^) to a *Har. marismortui *protein YP_136906; member of the ORC1/CDC-6 superfamily of NTPases involved in DNA replication
20	54% similarity (E 10^-13^) to halovirus ***φ***H1 repressor protein(AAV47198.1) with a winged helix DNA binding domain
21	66% similarity (E 10^-17^) to the *Hqr. walsbyi *PadR transcriptional regulator (CAJ51359.1)
31	similarity is to a *Har. marismortui *phage integrase (E 10^-66^) 45% ID (AAV47153 the ***λ ***bacteriophage recombinase family, pfam00589
35	DNA helicase? 62% similarity (E 10^-128^) to *Har. marismortui *protein (AAV47142) of the Cdc-46/Mcm family of DNA dependent ATPases.
39	68% similarity (E 0.05) to ArsR-like transcriptional regulator (CAJ51299) from *Hqr. Walsbyi *(92 amino acids long); the similarity being from amino acids 15–68 in ORF39 with 20–72 in CAJ51299
43	56% similarity (E 10^-37^), to halovirus HF1 protein (AAO61337.1) which may be a YonJ like, small subunit of the DNA polymerase, (COG1311)
	
48	54% similarity to *Listonella pelagia *phage phiHSIC small terminase subunit (YP_224235.1
49	43% similarity (E 0.01) to *Streptococcus pneumoniae *bacteriophage EJ-1 large terminase (CAE82121)
50	54% similarity (E 10^-77^) to the putative portal protein (NP_665924) of *Nab. magadii *virus ***φ***Ch1.
52	49% similarity (E 10^-13^) to the capsid protein gpD (AAM88683) of halovirus ***φ ***Ch1
53	48% similarity (E 10^-29^) to hp32 (CAA56442) of *Hbt. salinarum *virus ***φ***H and 47% similarity (E 10^-24^) to the capsid protein gpE (AAG32163) of halovirus ***φ***Ch1
51	51% similarity (E 10^-15^) to *Enterococcus faecium *glycosyl transferase (EAN10921). LPS biosynthesis protein.

The remaining 15 ORFs could have functions tentatively ascribed to them on the basis of amino acid similarity, (Table 2). We place them into three groups. (i) Those probably concerned with DNA replication, gene expression and possibly integration, i.e. ORFs 5, 6, 16, 20, 21, 31, 35, 39 and 43. (ii) Those proteins likely to be involved in virus assembly, i.e. ORFs 48, 49, 50, 52 and 53. (iii) Those proteins with other identifiable functions, i.e. ORF1.

### Nucleotide features

Nine direct repeats were observed greater than 13 nucleotides; the largest was 17 nucleotides, i.e. GGCGGCATCCAACTCGG repeated at positions 34076 and 34120. All of the repeats were located in putative ORFs and we can infer nothing of significance for them. A number of perfect and imperfect inverted repeat/stemloop structures were identified, often having loops 100 s–1000 s of nucleotides in size. One perfect palindrome is located at nucleotides _14226_GTCCGCTGGA/TCCAGCGGAC_14247 _in ORF31, the putative integrase gene. Another palindrome separated by 3 nucleotides (lower case) is _42048_ACTATCCGACtggGTCGGATAGT_42070_; again both are present in putative ORFs and their significance is unclear although the last palindrome is located 209 nucleotides from the 3' end of the genome. The BJ1 genome has a low incidence of CTAG and GATC sequences, just three of each of these palindromes being present. This incidence is low, both compared to the statistically expected incidence, (every 256 base pairs) and compared to the related tetramers CGAG and GCTC which were both found 36 times. CTAG and GATC sequences appear to be selected against by many haloviruses e.g. these palindromes are absent from the genomes of HF1, HF2, His2 and SH1 [6]. This selection pressure is thought to be due to the avoidance of restriction-modification systems in the host cells [26], and there is evidence that CTAG and GATC palindromes are used by haloarchaeal systems [27,28].

### Sequence heterogeneity

*Bam*HI digests of virion DNA gave rise to a fragment of about 3.5 kb, as judged by agarose gel electrophoresis, present in sub-stoichiometric amounts relative to the other bands, indicated by the black arrow in Fig 3c. This was fully sequenced and found not to fit into our genome assembly. Primers derived from this sequence were used with virus sequence primers and virus genomic DNA as a template. Products were observed with primers derived from the 3' end of ORF 32, suggesting that a minor subfraction of virion DNA did contain this *Bam*HI fragment. Sequencing showed that the site of insertion was at nucleotide 14790 in ORF 32 and showed that this part of ORF 32 was rich in CGX repeats, (Table 3). We have not yet been unable to derive PCR products defining either the location or 5' end of the insertion/substitution. Instead we have primer walked out from the defined 3' end of the insertion. ~8.7 kb of sequence has been determined [EMBL: AM491333] having a G+C content of 72.6 mol%, notably higher than the 64 mol% determined for the rest of the virus genome and close to that reported for *Hrr. saccharovorum *(71 mol%). Predicted ORFs have much higher homologies to known haloarchaeal proteins than the other viral ORFs, (Table 3). We think it most likely this sequence is derived from the host genomic DNA due to an integration/excision event.

**Table 3:** Predicted ORFs in the sequence inserted into ORF 32 and their highest BlastX matches. Nucleotide numbering is from the 5' end of the insertion sequence; nucleotide 8685 corresponds to nucleotide 14790 in the BJ1 genomic sequence. The sequence at the site of insertion was tgctcggtcgtcaa/CGACGCCGACGACGGCGA; lower case variant, upper case BJ1 ORF 32. Orfs are in the forward direction with respect to the virus genome unless indicated by a - sign. * indicates a truncated ORF because of incomplete sequencing (V10) or the insertion event itself (V1 and ORF32) aa indicates the number of amino acids.

ORF	Position		Size	Homologs (% Identity)
	Start	Stop	(aa)	

V10*	2	277	*	67% – ornithine cyclodeaminase *Natronomonas pharaonis *DSM 2160
V9-	749	351	132	36% – hypothetical protein VNG6157H *Halobacterium *sp. NRC-1
V8-	1910	843	355	70% – cell division protein pelota *Natronomonas pharaonis *DSM 2160.
V7-	3051	1936	371	28% – hypothetical protein NP4342A *Natronomonas pharaonis*
V6	3346	3753	135	38% – hypothetical protein rrnAC2062 *Haloarcula marismortui*
V5	3912	4685	257	38% – Alpha/beta hydrolase fold protein *Ralstonia eutropha *JMP134
V4	4747	5058	103	75% – hypothetical protein HQ2797A *Haloquadratum walsbyi *DSM 16790
V3-	7408	5900	502	73% – RtcB-like protein 1 *Natronomonas pharaonis *DSM 2160
V2-	7934	7503	143	61% – hypothetical protein NP3986A *Natronomonas pharaonis *DSM 2160
V1*	8326	8684	119*	64% – 3-hydroxy-3-methylglutaryl-coenzyme A reductase (HMG-CoA reductase) *Haloferax volcanii*
32*	8685	9059	*	100% Phage BJ1 hypothetical protein

## Discussion

Morphological criteria used for virus classification is outlined by the International Committee for Taxonomy of Viruses [7]. Virus BJ1 is an icosahedral head/tailed virus and as such is assigned to the order *Caudovirales *with examples infecting members of both the domains *Bacteria *and *Archaea*. BJ1 can also be assigned to the Bradley classification group B and might tentatively be assigned to the family *Siphoviridae *due to the apparent absence of a contractile tail, base plate and tail fibres and the presence of striations in the tail fibre. *If *we assume that this classification is phylogenetically justified then it could indicate that the *Caudovirales *originated before the divergence of the *Bacteria *and *Archaea *[29]. An alternative explanation is that the *Caudovirales *originally infected members of the domain *Bacteria *but that horizontal gene exchange from mesophilic *Bacteria *to the *Archaea *and the subsequent stabilisation of these genes in the *Archaea *allowed the *Caudovirales *to spread into the domain *Archaea *[Certainly we have detected diverse bacterial populations in the water of Lake Bagaejinnor, SH unpublished] [9].

As described in the Introduction, very few viruses infecting the domain *Archaea *have been described and as yet we have little idea as to the extent of virus diversity in this domain. The virus we describe here may not be a common or dominant member of the virus community infecting haloarchaea in saline waters. We screened for lytic virus particles forming plaques on archaeal lawns. These requirements for host culturability, good lawn formation and plaque formation are probably extremely restrictive. As pointed out by others, there is a genuine need to develop other isolation and culture techniques to study both the dominant virus populations and the true extent of archaeal virus variation in samples such as these – perhaps using a combination of electron microscopy and metagenomic sequence studies.

The GC content of BJ1 at 65 mol% is quite close to that reported for *Hrr*. spp *aidingense*, *lacusprofundi and saccharovorum*, varying from about 63–71 mol% [19,20]. The host strain for BJ1 clearly belongs to the genus *Halorubrum *having 98% 16SrRNA gene sequence identity to these *Halorubrum *species. Its precise taxonomic relationship to these species, in particular if it belongs to a new *Halorubrum *species is the subject of current studies.

Of the ORFs identified in BJ1 described in the results, all of the statistically significant matches are recorded, (Table 2). Six of the ORFs (9, 20, 50, 52, 53, 55) are most closely related to the haloarchaeal temperate, isometric head/contractile tail viruses ***φ***Ch***1 ***[13] and the intensively studied, ***φ***H [30]. These two viruses are closely related to each other, the completed genome of ***φ***Ch***1 ***shows 97% homology to the genome of ***φ***H, which is about 60% complete. ORF 43 is most closely related to a gene from the haloarchaeal isometric head/contractile tail virus HF1. There are no similarities with the ORFs from either the spindle (His1, His2) or icosahedral (SH1) shaped haloarchaeal viruses described in the Introduction. The most significant matches were ORFs 16, 31, 35, which are almost certainly the origin of replication complex, integrase and helicase functions respectively of the virus, having highly significant matches to full length proteins in *Har. marismortui*. ORF50 was also closely related to the putative portal protein (NP_665924) of *Nab. magadii *virus ***φ***Ch1.

Speculatively, almost all ORFs are in the forward strand in the same direction consistent with a rolling circle mechanism of DNA replication. The 7 ORFs on the reverse strand including the integrase may be poorly expressed. A few ORFs had GTG starts (but with good RBS sequences) and the other ORFs lacked RBS sequences altogether, presumably both coding features control/reduce expression levels. The fact that putative *Int *gene is coded for on the minor strand with no RBS and that it overlaps with ORF 30 on the major strand may indicate that its expression is tightly controlled; perhaps most infections are lytic with a small proportion of lysogenic events. The suggestion of operons indicated in Fig 4 is also entirely speculative and based on the presence of overlapping stop and start signals, one run of ORFs from 43–48 has no RBS at all. Proteins with putative functions involved in DNA replication and transcription are found in ORFs 1–43, putative structural proteins are found after ORF48 consistent with early and late expression of operons.

Although BJ1 stocks are clonal in origin, the genomic DNA preparation is obviously and necessarily derived from a virus pool. Genome sequence projects often therefore give rise to heterogeneous sequences. We found one substantial region of heterogeneity in ORF 32 at nucleotide 14790 involving either a large insertion or more probably a substitution event (since terminally redundant virus genomes usually package genomes in a 'head full' mechanism). To distinguish between these possibilities requires more sequencing. The variant sequence probably involves the acquisition of host derived DNA since the GC content is higher (72.6%) than that of the virus (64.8%) and close to that reported for *Hrr. saccharovorum *(71%). Obviously this insertion/substitution has taken place about 300 nucleotides away from the putative integrase gene. The integrase gene in viruses is often the site of insertion as well. We speculate that this variant sequence in the virus population is the result of an integration/excision event (possibly aberrant) during the virus infection to prepare genomic DNA. This may indicate that BJ1 is a lysogenic virus; plaques were certainly turbid consistent with this suggestion but further experiments will be required to prove it. Whether the virus population with this variant sequence is viable will also require further studies. Certainly virus populations with insertions and or substantial genomic deletions can be viable or at least rescued by functional virus genomes.

Many interesting features remain to be discovered about the BJ1 virus. Optimal growth conditions for this virus need to be established and its host range determined. This will facilitate studies on its environmental stability, patterns of transcription, protein functions, lysogenic potential and the viability of the variant virus. Assignment of protein functions to ORFs which cannot be assigned any function based on sequence identity is probably easier using a virus as a model than any other genome. A systematic effort on this front will reduce the number of unclassified ORFs that metagenomic and archaeal sequencing projects so often throw up.

## Methods

### Cultivation of prokaryotes from environmental samples

Isolates were grown on a modified Classic Halophile Medium (mCHM) broth, [31]. This was made in two components; component 1 contains 1% (w/v) yeast extract, 0.75% (w/v) casamino acids, 0.248% (w/v) KCl and 0.3% (w/v) trisodium citrate; component 2 contains 0.162% (w/v) Na_2_B_4_O_7_, 0.084% (w/v) NaBr, 7.116% (w/v) MgCl_2_.7H_2_O, 13% (w/v) NaCl, 4.56% (w/v) Na_2_SO_4_, 0.062% (w/v) NaHCO_3 _and 0.036% (w/v) Na_2_CO_3_, pH 8.0. Both components were autoclaved separately and mixed once cooled to 60°C, then stored at room temperature. 2% (w/v) agar was added to component 1 if required to make mCHM agar plates, while 0.7% (w/v) agar was added to component 1 to make soft top agar. Prokaryotes were cultivated from brine, salt or sediment samples. Brine was filtered on site through sterile 0.45 μm membrane filters in a 250 ml capacity polycarbonate filter unit (Sartorius) using a Nalgene hand pump until flow stopped. Membrane filters were immediately placed in cold sterile stabilisation buffer (10 mM Tris-HCl, pH 8.0, 1 mM EDTA, 2 M NaCl) and agitated to resuspend the cells. Filtered waters were placed in sterile falcon tubes. Samples were placed immediately on ice until they could be stored at -20°C, usually within 6 hours of collection. Either, cell suspensions from agitated filters were serially diluted and plated onto mCHM agar plates, or about 0.5 g sediment and salt crust was resuspended in 0.5 ml of mCHM and serial dilutions plated onto the mCHM agar plates. These were incubated for two months at 37°C and were periodically checked for the appearance of new colonies which were picked and grown on fresh plates. Sub-culturing was continued on the same medium until purity was achieved. Isolated colonies were then grown in mCHM broth to an OD_695 _of 2 to 4, and maintained on sterile beads at -80°C for long-term storage in mCHM broth with 30% (v/v) sterile glycerol.

### Identification of haloarchaeal isolates by 16S rRNA gene sequencing

Pure cultures, see above, were lysed in 100 μl nanopure water and boiled for 10 min. Cell debris was pelleted by centrifugation at 13 000 × g for 10 min. 1 μl cell lysate was used in a PCR reaction containing (75 mM Tris-HCl, pH 8.8, 20 mM (NH_4_)_2_SO_4_, 0.01% (v/v) Tween 20), 0.2 mM dNTPs, 3 mM MgCl_2_, 20 *p*mol forward primer, 20 *p*mol reverse primer, 2.5 U *Taq *polymerase and nanopure water to a final volume of 50 μl. To amplify the 16S rRNA genes, the *Archaeal *domain specific primer 27Fa, 5'-TCY GGT TGA TCC TGS CGG-3', [32] and rP1 5'-ACG GHT ACC TTG TTA CGA CTT-3', [33] were used. Reaction conditions were: 95°C for 2 min, followed by 30 cycles of 95°C for 30 s, 50°C for 40 s and 72°C for 2 min, followed by 10 min extension time at 72°C. PCR products were purified using the QIAquick PCR Purification Kit (Qiagen) and stored at -20°C until required. DNA sequencing, also see below, was done by Lark Technologies, Cambridge UK using 27Fa and rP1 primers described above (corresponding to nucleotides 27–1492 with *E. coli *as the reference sequence). The DNA sequences were analysed using the BLASTN homology search program [34], which is available at the National Centre for Biotechnology Information to identify close matches.

Strains were placed on a phylogenetic tree using Molecular Evolutionary Genetics Analysis (MEGA) version 3.1 [35], using the Jukes and Cantor nucleotide substitution model for sequence alignment and the Neighbour-Joining method of tree inference. The support for each node was determined by assembling a consensus tree of 500 bootstrap replicates.

### Isolation of haloarchaeal virus by plaque assays

Haloarchaeal strains identified as described above were grown in soft top agar. mCHM bottom agar plates were overlaid with mCHM soft top agar containing 0.75% (w/v) agar, kept molten in a 55°C water bath until required. 300 μl of the haloarchaeal strain (OD approximately 0.2 at 695 nm, avoiding absorbance by the archaeal pigments) was added to 3 ml agar cooled to approximately 50°C and mixed. This was immediately poured on top of the bottom agar and left to set. The plates were carefully inverted and incubated in a sealed bag at 37°C for a week or longer. If good lawns were formed the strain was used to isolate haloarchaeal virus as follows: 10 ***μ***l of Bagaejinnor lake water passed through both a 0.45 and 0.22 μm filter (both from Millipore) was added to 1 ml cell culture and incubated at 37°C in an orbital shaker at 150 rpm overnight. The culture was plated in soft top agar as described and the resulting lawns checked for the appearance of lytic plaques. Single plaques selected for purification were picked with a sterile toothpick. Virus particles were then resuspended in 100 μl mCHM broth; this was then used to infect the host as previously described. This process of plaque purification was repeated twice to ensure that the virus samples were pure. Virus particles remained stable in mCHM broth when placed at 4°C for at least 1 year.

### Transmission electron microscopy

5 ***μ***l of the virus sample was adsorbed onto glow discharged, carbon coated pioloform grids and fixed in glutaraldehyde vapour for 3 min. Excess sample was blotted from the grid using filter paper. Salts were removed by washing with distilled water. The sample was visualised by negative staining using 1% (w/w) uranyl acetate and viewed on a JEOL 1220 transmission electron microscope fitted with a SIS Megaview III digital camera system. Captured Images were viewed and analysed using the Image J program [36].

### Viral nucleic acid extraction

Attempts to purify virus nucleic acid from infected liquid cultures were unsuccessful. Accordingly 30 μl of virus stock (~10^6^pfu/ml) were added to 300 μl of host cell culture (OD approximately 0.2 at 695 nm). Virus particles were left to adsorb onto the host cells for 15 min at room temperature, mixed with soft top agar and poured and incubated as described above to give agar plates with a high density of virus plaques. 0.5 ml halovirus diluent [60% (v/v) of a salt solution containing; 0.3% (w/v) KCl, 0.162% (w/v) Na_2_B_4_O_7_, 0.084% (w/v) NaBr, 7.116% (w/v) MgCl_2_.7H_2_O, 13% (w/v) NaCl, 4.56% (w/v) Na_2_SO_4_, 0.062% (w/v) NaHCO_3 _and 0.036% (w/v) Na_2_CO_3_; 29% (v/v) H_2_0; 1% (v/v) 1 M Tris pH 7.2; 10% (v/v) glycerol] was added to each plate and the virus harvested by scraping off the soft top agar and homogenising by vortexing for 30 s. Agar and cell debris was pelleted by centrifugation at 10 000 rpm for 20 mins. The supernatant was transferred to a fresh clean tube. To increase the yield of virus particles, the pellet was resuspended in 2 ml halovirus diluent and the previous steps of homogenisation and centrifugation were repeated. Combined supernatants were passed through a 0.45 μm filter and then a 0.22 μm filter to further remove agar and cell debris. To remove any exogenous non-virus nucleic acids DNase I and RNase A were each added to a final concentration of 1 μg/ml and the sample left at room temperature for 30 min.

Virus particles were precipitated by the addition of 1/8 volume polyethylene glycol (PEG) 6000 solution (2.5 M NaCl, 20% (w/v) PEG 6000) and left to incubate for 15 min at room temperature. Virus particles were pelleted by centrifugation at 13 000 × g for 5 min. The supernatant was carefully removed and the pellet resuspended in 100 μl phosphate buffered saline (0.8% w/v NaCl, 0.121% w/v K_2_HPO_4 _and 0.034% w/v KH_2_PO_4_). To extract genomic nucleic acid from the virus, the pellet was mixed with an equal volume of phenol chloroform and centrifuged for 30 s. The top nucleic acid containing aqueous layer was transferred to a fresh tube. Excess phenol chloroform was removed by ether extraction. The nucleic acid was ethanol precipitated, redissolved in 20 μl Tris-EDTA, pH 8.0 and left to rehydrate at 4°C overnight. An extraction from 20 plates typically yielded 1–2 ***μ***g nucleic acid.

### Genome characterisation and sequencing

1 ***μ***g virus nucleic acid was treated with either excess DNase I (NEB), RNase A (Sigma) or Exonuclease III (NEB) in the manufacturers reaction buffer and incubated at 37°C for 10 min, 60 min or 30 min respectively. Reactions were electrophoresed on Tris-Acetate-EDTA (TAE) agarose gels and stained with SYBR green. Viral nucleic acids were ran on a 1% agarose pulse field gel (BioRad) in 0.5× TBE buffer at 14°C in a CHEF DR-II apparatus (BioRad). The run time was 22 h with a voltage gradient of 6 V/cm and a linearly ramped pulse time of 50 to 90 s at an angle of 120°.

BJ1 genomic DNA was digested with *Bam*HI (giving approximately 20 fragments ranging in size from 100 bp to 5 kbp, and cloned into *Bam*HI-digested pUC18*Not*I vector [37]. Resulting clones were sequenced using vector-specific oligonucleotide primers pUCF, 5'-GTTTTCCCAGTCACGACGTTG-3' and pUCR, 5'-CACAGGAAACAG CTATGACC-3'; these sequences were used to design further primers to primer walk across the clones. The high G+C content (~65 mol%) of the initial sequences was used to identify restriction enzymes that would likely cut the phage genome to give smaller (on average 500–1000 bp) fragments. Secondary libraries of *Sst*I and *Xho*I fragments were created in pUC18*Not*I and representative clones of these libraries were sequenced using pUCF and pUCR and subsequent primer walking. Finally the remaining gaps were filled by designing primers to the ends of the larger contigs, orientating these contigs by PCR using phage genome as template, and then primer walking out from the contigs using the PCR amplified products as sequencing template. The genomic sequence was assembled using the Lasergene SeqMan 7.0 program (DNAStar). Final coverage of the genome was 4-fold with the majority sequenced on both of the strands or, where bidirectional sequencing was impractical, with multiple sequence runs on the same strand.

### Bioinformatics

Potential ORFs were assigned using the programs FGENESB [38] and GeneMark.hmm v2.5a [39]. tRNA sequences were identified using the tRNAscan-SE program in [40]. Translations of potential ORF sequences to amino acids were made with the SeqBuilder program (DNAStar). Statistics for each of the ORFs were calculated using the program ProtParam [41].

GC skew was calculated using the online base composition tools at [42]. BLAST (blastp and tblastn) and PSI-BLAST [43] were used to search for possible homologies to known proteins, or proteins predicted by translation of the unannotated DNA sequence in GenBank. Inverted repeats in the DNA sequence were identified using Einverted [44] and PALINDROME [45]; direct repeats were located using Palim [46].

## Authors' contributions

Eulyn Pagaling collected samples on the field trip, did all of the culture and virus isolation work and analysed and interpreted data and revised the manuscript. Richard Haigh determined and analysed much of the genomic sequence data and revised the manuscript. W Grant, D Cowan, B Jones, Y Ma, and A Ventosa, participated in the experimental design, collected samples on the field trip analysed and interpreted data and revised the manuscript. Shaun Heaphy participated in the experimental design, collected samples on the field trip, supervised laboratory work, analysed and interpreted data and wrote the manuscript. All authors read and approved the final manuscript.
